# Experimental evaluation of antipyretic and analgesic activity of aspartame

**DOI:** 10.4103/0253-7613.75683

**Published:** 2011-02

**Authors:** Sapna Pradhan, U. H. Shah, A. Mathur, S. Sharma

**Affiliations:** Department of Pharmacology, Army College of Medical Sciences, Delhi. E-mail: drsapnapradhan@gmail.com; 1 BJ Medical College, Ahmedabad, India

Sir,

It has been reported that aspartame is biologically active and appears to relieve pain and induce mild antithrombotic effects in humans, and decrease fever in animals.[1] There is, however, a paucity of data about the biological effects of aspartame, which prompted us to undertake the present study, with a view to investigate the possible antipyretic and analgesic effects of aspartame and its comparison with conventional drugs.

Albino Wistar rats of either sex (age 3-4 months, weight 200 g) were used to study the antipyretic effect, while adult mice of either sex, weighing 20-30 g, were used to study the analgesic effect. The care and maintenance of animals were as per the approved guidelines and the study was approved by the Institutional Animal Ethics Committee. Aspartame was obtained from Zydus Cadila (Ahmedabad, India).

The antipyretic effect was evaluated by a 2-hour test using TAB vaccine (0.3 mL, i.p.). Rectal temperature was recorded using a clinical thermometer for a period of 2 minutes, both in basal state and at 30 minutes interval for 2 hours after the administration of vaccine. The animals were divided into five groups of 10 animals each. Group I received 0.3 mL distilled water (control). Groups II, III and IV received aspartame in a dose of 2, 4 and 8 mg/kg, respectively. Group V was administrated paracetamol (220 mg/kg). The reduction in mean rise of temperature was noted.

The analgesic effect was studied by acetic acid induced writhing method.[2] Mice were divided into five groups of 10 animals each. Group I received 0.3 mL distilled water (control). Groups II, III and IV received aspartame in doses of 2, 4 and 8 mg/kg, respectively. Group V was given diclofenac 1 mg/kg by intraperitoneal route, 15 minutes prior to acetic acid injection. After intraperitoneal injection of 3% aqueous acetic acid (300 mg/kg), the mice were observed under a bell jar for a period of 20 minutes and the number of writhing episodes was noted. An average value for each group was calculated and subsequently used for comparison. In addition, protection provided by the test drugs was also calculated using the formula

$$\%\;protection\;=100\;–\left(\frac{number\;of\;writhes\;in\;\exp erimental\;group\;\times \;100\;}{number\;of\;writhes\;in\;control\;group\;}\right)$$

Table 1 shows the effect of aspartame on rectal temperature of rats following administration of TAB vaccine. Aspartame showed a statistically significant (*P* < 0.001) antipyretic effect at all the doses used and this was comparable with paracetamol. Table 2 shows the effect of aspartame and diclofenac on acetic acid induced writhing episodes in mice. Aspartame in doses of 4 and 8 mg/kg showed statistically significant (*P* < 0.01) protection against writhing. Diclofenac, a potent analgesic compound, produced highly significant (*P* < 0.001) effect on writhing in doses of 1 mg/kg, i.p.

**Table 1:** Mean rise of rectal temperature (°F) in rats

*Group (treatment)*	*Time after TAB vaccine administration (minutes)*			
	*30*	*60*	*90*	*120*
I (Distilled water, 0.3 mL, i.p.)	0.24 ± 0.1	1.16 ± 0.33	1.46 ± 0.17	1.50 ± 0.42
II (Aspartame, 2 mg/kg, i.p.)	0.0 ± 0	0.38 ± 0.21	0.44 ± 0.16**	0.44 ± 0.22
III (Aspartame, 4 mg/kg, i.p.)	0.0 ± 0	0.22 ± 0.12*	0.30 ± 0.13**	0.34 ± 0.18
IV (Aspartame, 8 mg/kg, i.p.)	0.0 ± 0	0.18 ± 0.10*	0.24 ± 0.11**	0.26 ± 0.19
V (Paracetamol, 220 mg/kg, i.p.)	0.0 ± 0	0.04 ± 0.03*	0.08 ± 0.06**	0.10 + 0.07*

Each value represents mean ± SEM (n = 10);

* *P* < 0.05,

** *P* < 0.001 as compared to control (student’s unpaired “t” test)

**Table 2:** Effect of aspartame and diclofenac on acetic acid induced writhing episodes in mice

Group (treatment)	No. of writhing episodes	Inhibition (%)
I (Distilled water, 0.3 mL, i.p.)	30.2 ± 1.05	—
II (Aspartame, 2 mg/kg, i.p.)	24.8 ± 1.72	17.88
III (Aspartame, 4 mg/kg, i.p.)	23.6 ± 1.75*	21.85
IV (Aspartame 8 kg/mg, i.p.)	22.8 ± 1.89*	24.50
V (Diclofenac, 1.mg/kg, i.p.)	16.4 ± 1.35**	45.70

Each value represents mean ± SEM (n = 10);

* *P* < 0.01,

** *P* < 0.001 as compared to control (student’s unpaired “t” test)

In our study, we found that aspartame has highly significant antipyretic effect in doses of 2, 4 and 8 mg/kg. To our knowledge, there are no published reports of experimental evaluation of antipyretic effect of aspartame in this model. We also found that in doses of 4 and 8 mg/kg, aspartame showed a significant analgesic activity. Another study has shown that oral administration of aspartame (2-16 mg/kg) significantly increased the pain threshold against acetic acid induced writhing in mice.[3] In yet another study, it has been shown that aspartame attenuates mechanical allodynia in high doses (50 mg/kg).[4] Since this study was conducted in carrageenan-induced monoarthritis model, it appears that differences in the experimental models of evaluation of analgesic activity may account for the variation in effective doses. Interference with rheumatoid factor activity has been proposed to alleviate the pain and immobility resulting from chronic inflammation of joint.[5] In the acetic acid induced writhing method, nociceptive response involves release of endogenous substrates like bradykinin and prostanoids.[6] It is speculated that the antagonism of these chemicals may underlie the pain-relieving mechanism of aspartame. However, the exact mechanism of analgesic action of aspartame can be investigated by further pharmacological studies to overcome the limitations of current work.

To conclude, aspartame is an intense nutritive sweetener, which is widely used by diabetic patients and overweight persons. Our study has demonstrated an antipyretic effect of aspartame and confirmed its analgesic effect in an experimental model. Further studies on biological effects of aspartame are required to explore its therapeutic potential as an antipyretic and analgesic agent.
